# Virtual Reality Analgesia for Children With Large Severe Burn Wounds During Burn Wound Debridement

**DOI:** 10.3389/frvir.2020.602299

**Published:** 2020-12-10

**Authors:** Hunter G. Hoffman, David R. Patterson, Robert A. Rodriguez, Raquel Peña, Wanda Beck, Walter J. Meyer

**Affiliations:** 1 Department of Mechanical Engineering, College of Engineering, University of Washington, Seattle, WA, United States; 2 Department of Psychology, University of Washington, Washington, ME, United States; 3 Department of Radiology, University of Washington, Seattle, WA, United States; 4 Department of Rehabilitation Medicine, University of Washington, Seattle, WA, United States; 5 University of Texas Medical Branch at Galveston, Galveston, TX, United States; 6 Shriners Hospitals for Children Galveston, Galveston, TX, United States

**Keywords:** virtual reality, pain, pediatric burn injuries, analgesia, burn, opioid

## Abstract

The objective of this study was to compare the effect of adjunctive virtual reality vs. standard analgesic pain medications during burn wound cleaning/debridement. Participants were predominantly Hispanic children aged 6–17 years of age, with large severe burn injuries (TBSA = 44%) reporting moderate or higher baseline pain during burn wound care. Using a randomized between-groups design, participants were randomly assigned to one of two groups, (a) the Control Group = pain medications only or (b) the VR Group = pain medications + virtual reality. A total of 50 children (88% Hispanic) with large severe burns (mean TBSA > 10%) received severe burn wound cleaning sessions. For the primary outcome measure of worst pain (intensity) on Study Day 1, using a between groups ANOVA, burn injured children in the group that received virtual reality during wound care showed significantly less pain intensity than the No VR control group, [mean worst pain ratings for the No VR group = 7.46 (SD = 2.93) vs. 5.54 (SD = 3.56), *F*_(1,48)_ = 4.29, <0.05, MSE = 46.00]. Similarly, one of the secondary pain measures, “lowest pain during wound care” was significantly lower in the VR group, No VR = 4.29 (SD = 3.75) vs. 1.68 (2.04) for the VR group, *F*(_147_) = 9.29, < 0.005, MSE = 83.52 for Study Day 1. The other secondary pain measures showed the predicted pattern on Study Day 1, but were non-significant. Regarding whether VR reduced pain beyond Study Day 1, absolute change in pain intensity (analgesia = baseline pain minus the mean of the worst pain scores on Study days 1–10) was significantly greater for the VR group, *F*_(148)_ = 4.88, *p <* 0.05, MSE = 34.26, partial eta squared = 0.09, but contrary to predictions, absolute change scores were non-significant for all secondary measures.

## INTRODUCTION

Excessive acute pain during medical procedures is a common problem for a wide range of patient populations. As an extreme example, children recovering from large severe burn injuries frequently receive painful burn wound cleaning procedures several times a week, often daily, during hospitalization. Acute procedural pain during burn wound debridement is often difficult to control, despite pre-medicating patients with powerful traditional pharmacologic analgesics before wound care (Hoffman et al., 2019b). Opioid analgesic side effects restrict dose levels and limit the amount of pain reduction from medications alone (Cherny et al., 2001; Malchow and Black, 2008; Clark et al., 2017; Ballantyne, 2018; Dunwoody and Jungquist, 2018). Moreover, tolerance to the medication can also reduce analgesic effectiveness, especially for burn patients who have the same painful wound debridement procedure repeated frequently (Bittner et al., 2015, see also Ballantyne, 2018). Undermedication of acute pain has been a common and longstanding concern (Melzack, 1990; Krane, 2019). Many non-western countries have little or no availability of opioid analgesics (Berterame et al., 2016), and such agents are becoming more tightly regulated in the United States (Davis et al., 2018). Although wound cleaning is often crucial to recovery and is done to avoid infection, wound care of an extensive burn is unusually painful (Hoffman et al., 2019b). In addition to the humanitarian objectives of reducing suffering in children, there is growing awareness that excessive pain can lead to long term medical complications such as posttraumatic stress disorder and chronic pain (Ehde et al., 1999; McGhee et al., 2011; Schwaller and Fitzgerald, 2014; Rosenberg et al., 2015; Peña et al., 2017; Nelson et al., 2019). Furthermore, effective control of acute pain can help offset medical costs (Montgomery et al., 2000; Lang and Rosen, 2002).

Anxiety, fear, anticipation of pain (Hemington et al., 2017; Fields, 2018), stimulus response conditioning/learned aversion (Birnie et al., 2017), memories of previous painful events (Noel et al., 2015), and other noxious psychological factors can amplify a patient’s pain during medical procedures (Melzack and Wall, 1965). Fortunately, psychological interventions can help reduce acute pain, and are often used adjunctively with traditional pain medications. Immersive virtual reality (VR) is proving to be an unusually effective non-drug pain control technique. VR has been shown to reduce pain and anxiety during burn care (Hoffman et al., 2000a, 2001, 2014; Sharar et al., 2007; Carrougher et al., 2009; Soltani et al., 2018), dental procedures (Atzori et al., 2018a), venipuncture (Gold et al., 2006, 2007; Atzori et al., 2018b; Gold and Mahrer, 2018), blunt force trauma (Hoffman et al., 2009), and a number of other painful medical procedures (see Hoffman et al., 2000b, 2011, 2019a, 2020; Keefe et al., 2012; Garrett et al., 2014 for reviews). Several studies have explored the effectiveness of VR pain reduction during burn wound cleaning of burn patients treated for smaller injuries [i.e., <10% Total Body Surface Area (TBSA); (Faber et al., 2013; Garrett et al., 2014; Jeffs et al., 2014; Khadra et al., 2020)]. Using a within-subject design study of pain during wound care, US soldiers being treated for combat-related burn injuries (21% TBSA) reported significant reductions in pain during VR, and significantly more fun during wound care (Maani et al., 2011; see also Sharar et al., 2016).

Despite the growing body of encouraging research showing the pain reducing potential of VR (Trost et al., 2020), it is possible that this technique will not be effective if the patient’s pain is too intense. Although the mechanism of how virtual reality reduces pain is not well-understood, researchers propose that VR reduces pain primarily *via* attention distraction (Hoffman et al., 2000a,b). Pain requires conscious attention to process nociceptive signals (Eccleston and Crombez, 1999) and attention is a limited mental capacity (Kahneman, 1973). VR and pain are competing for the brain’s limited attentional resources in what is essentially a divided attention task. The patient’s brain is inundated with multi-sensory information from the VR system (primarily through the visual system). This leaves their brain with less attention available to process incoming nociceptive signals from pain receptors in the patient’s skin. The more attention grabbing the VR, the less attention is available to process pain perception information. But the reverse may also apply. The more intense the patient’s pain, the harder it may become to lure the patients spotlight of attention away from their pain and into the virtual world.

McCaul and Malott (1984) have speculated that distractions will work best during mild to moderate pain, because, at higher pain intensities, they predict that severe to excruciating pain intensity could attract enough attention to render distraction useless. Whether Virtual Reality can help patients who are currently experiencing extreme levels of pain, has been largely unexplored.

A recent within-subject pilot study of children hospitalized for treatment of large severe burns suggests that VR can work in this challenging Intensive Care Unit (ICU) context. In their recent study, 48 pediatric patients with large burn injuries (40% TBSA) received VR vs. No VR during different portions of the same wound care session, in an ICU tank room setting. Patients reported significantly lower pain intensity during burn wound care when they were in VR (SnowWorld) vs. during No VR (pain medications only) and patients continued to report the predicted pattern of less pain and more fun during multiple wound care sessions (Hoffman et al., 2019b).

As the next step, using a randomized between-groups design, the current study compares pain during wound care with one group receiving adjunctive virtual reality and another group receiving No VR (standard of care pain medications).

## MATERIALS AND METHODS

The current study was conducted between July 2015 and December 2017, in accordance with the Declaration of the World Medical Association (www.wma.net), with approval from the University of Texas Medical Branch Ethics Committee Internal Review Board, and all participants and their parents/legal guardians provided their written informed consent/assent in accordance with the Declaration of Helsinki.

Patients in the current study were treated at a large regional children’s burn center that specializes in treating pediatric patients with unusually large severe burn injuries. Many of the patients (88%) were transported to the burn center in Galveston Texas from Latin American countries and treated with support from humanitarian philanthropies in both the USA and Mexico. Such children received in house treatment in Galveston and returned to their home countries after discharge.

### Inclusion Criteria

Children were included in the current study if they were cooperative and able to answer the questionnaire, did not have a history of previous psychiatric (DSM-5 Axis I) disorder(s), did not have delirium, psychosis, or any form of organic brain disorder, were able to speak and understand English or Spanish, if they reported at least moderate worst pain intensity (worst pain ratings of 5 or higher on a 0 to 10 rating scale) during a No VR baseline wound care session, if they were admitted to the regional burn center in Texas and completed at least one baseline wound care session and one study day wound care session. Children were excluded from the study if the amount of skin burned was less than 10% TBSA, if they were unable to complete the study measures, if the patient did not require at least one study day wound cleaning session, if the patient had a previous history of psychiatric (DSM-5 Axis I) disorder(s), was currently showing evidence of delirium, psychosis, or an organic brain disorder, if the patient was not able to speak and understand English or Spanish, had a previous medical history of significant endocrine, cardiac, neurologic, respiratory, metabolic, genitourinary, or gastrointestinal impairment, if the patient was being treated for alcohol or drug withdrawal, was developmentally disabled, was less than 6 years old, over 17 years old, unable to use the VR equipment due to burns (e.g., burned eyes), or had a previous history of extreme sensitivity to severe motion sickness.

### Apparatus

Head and face burns are common for this patient population and can make it challenging or impossible for severe burn patients to wear a conventional VR helmet. Furthermore, conventional VR helmets are designed to be worn by a person that is either standing or sitting, whereas many burn patients are laying on their back on a shower table during wound care in the ICU. In the current study, a battery powered VR system was brought into the wound care room on an Anthro medical cart. Instead of wearing a head mounted display, a special robot-like arm goggle suspension system mounted to the cart allowed researchers to position the VR goggles near the patients head, without touching the patient, so the patient could look into the VR goggles without wearing a helmet. The goggles used were MX90 VR goggles, from NVISinc.com, with 90 degrees diagonal field of view, per eye (unusually wide peripheral vision at the time the study was initiated in 2015), and 1,280 × 1,024 pixels resolution per eye. The VR goggles largely obscured the patients view of the wound care and substituted pleasant computer simulated imagery of SnowWorld viewed through the VR goggles. In SnowWorld, patients floated slowly through a 3D canyon, with an icy river flowing at the bottom of the canyon. They could see Snowmen, igloos, wooly mammoths, and flying fish in the virtual world. When interviewing patients and nursing staff when designing SnowWorld, some burn patients reported that wound care and the associated pain reminded them of the fire in which they were originally burned. SnowWorld was designed to be the antithesis of fire, to help patients avoid their pain during wound care, and to help patients avoid thinking about fire during wound care, in a simple environment that is easy to render, attention grabbing (e.g., interactive) but non-nauseogenic with passive navigation, and canyon walls that discourage wild changes in viewpoint (Bloemink et al., 2006, p. 106). Patients could interact with virtual objects by moving and left clicking a wireless computer mouse to throw snowballs in VR. The virtual objects reacted with special effects and sound effects when hit by the patient’s virtual snowballs. Snowmen disappeared with a white puff, flying fish froze into ice in mid-air, and shattered into ice pieces with sound effects when hit by several snowballs, and animated wooly mammoths trumpeted angrily when hit by several snowballs. Although they were not able to wear earphones because of their burns, patients heard 3D sound effects mixed with music *via* battery powered Bose speakers positioned on the cart. These sound effects helped provide converging evidence from multiple senses (e.g., what they see and hear), to help increase patients’ illusion of “being in a place” in the computer-generated world.

The VR system was approved for use in the ICU and was periodically inspected by clinical engineering and infection control. Cleanliness of the VR equipment was also periodically monitored by infection control at the treating hospital. In compliance with institutional infection control, clear disposable plastic was used to further reduce contact between the patient and the VR eyepieces and mouse, and the disposable plastic coverings were thrown away after each use. Chemical disinfectants were used to carefully clean the VR equipment after each use, and an ultraviolet radiation UV sterilization lamp was also sometimes used. For infection control, cotton swabs were periodically rubbed on the VR equipment (especially the goggles and mouse) to detect if any pathogenic bacteria were present. The researchers sent culture samples to the microbiology testing laboratory at the treating institution. All post-cleaning tests were returned with no evidence of pathogens.

## MEASURES

The current study measures included “Time spent thinking about pain during burn wound care,” (a measure of the cognitive component of pain), pain unpleasantness (a measure of the emotional component of pain), worst pain (a measure of the pain intensity), and lowest pain.

Previous studies have shown that pain unpleasantness and worst pain are separate components of the pain experience that are sometimes differentially activated and can activate different parts of the brain (Rainville et al., 1997; Rainville, 2002). For example, increased emotional pain (e.g., pain unpleasantness) has been associated with increased activity of the anterior cingulate cortex, and increases in the sensory component of pain intensity (worst pain) are associated with increased activity in the primary and secondary somatosensory cortex (Rainville et al., 1997; Rainville, 2002).

Using Graphic Rating Scales (GRS), shortly after the wound cleaning session, all patients retrospectively rated their worst pain during wound care during a “no VR” baseline wound care session. Similarly, patients retrospectively rated their worst pain shortly after their wound care session on each study day. The GRS is a horizontal line labeled with the numbers 0 to 10, with word descriptors under the numbers.

Shortly after each wound cleaning session was completed (after the baseline day, and after each study day), patients were given the following instructions prior to giving six separate ratings: “Please indicate how you felt during the wound cleaning session just completed by making a mark anywhere on the line. Your response doesn’t have to be a whole number.” A pictorial example of the labeled graphic rating scale was shown for each question. Rate your WORST pain during wound care. 0 = no pain at all, 1−4 = mild pain, 5−6 = moderate pain, 7−9 = severe pain, 10 = excruciating pain.

Rate your WORST PAIN intensity during that wound care:


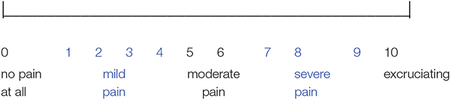


How much TIME did you spend thinking about pain? 0 = none of the time, 1−4 = some of the time, 5 = half of the time, 6−9 = most of the time, 10 = all of the time. How UNPLEASANT was the most recent wound session? 0 = not unpleasant at all, 1−4 = mildly unpleasant, 5−6 = moderately unpleasant, 7−9 = severely unpleasant, and 10 = excruciatingly unpleasant. Rate your LOWEST pain during wound care. 0 = no pain at all, 1−4 = mild pain, 5−6 = moderate pain, 7−9 = severe pain, 10 = excruciating pain. How much FUN did you have during wound care? 0 = No fun at all, 1−4 = mildly fun, 5−6 = moderately fun, 7−9 = pretty fun, 10 = extremely fun. How much did you feel NAUSEA (sick to your stomach) during the most recent wound care session? 0 = no nausea, 1−4 = mild nausea, 5−6 = moderate nausea, 7−9 = severe nausea, 10 = vomit.

On Study Day 1, patients in the VR Group were asked two extra questions (adapted from Slater et al., 1994). How much did you feel like you WENT INSIDE the VR SnowWorld game? 0 = I did not feel like I went inside at all, 1−4 = mild sense of going inside, 5−6 = moderate sense of going inside, 7−9 = strong sense of going inside, 10 = I went completely inside the SnowWorld game. How REAL did things in the computer world seem to you? 0 = completely fake, 1−4 = somewhat real, 5−6 = moderately real, 7−9 = very real, 10 = indistinguishable from a real object. All text was available in both English and Spanish.

The Graphic Rating Scale (GRS) is one of the most reliable and valid subjective measures of pain (Jensen and Karoly, 2001; Jensen, 2003; Williamson and Hoggart, 2005). “The visual analog and graphic rating scales were more sensitive than the traditional simple descriptive pain scale. Most patients could readily use visual analog and graphic rating scales despite having no previous experience” (Scott and Huskisson, 1976, p. 175). The Graphic Rating Scale has also been validated for patients aged 8 and older (Tesler et al., 1991).

### Experimental Design

In the current between-groups study design, pediatric patients with large severe burn injuries were randomized to one of two groups, using random sequences generated at random.org and using blocked randomization (e.g., “No VR Group, Yes VR Group”; “Yes VR Group, No VR Group,” etc.) to help equalize the final group sizes.

### Pre-procedural Pain Medications

Each subject received standard pre-procedure pharmacologic analgesia, according to the published guidelines used at this hospital (Ratcliff et al., 2006; Herndon, 2018, p. 694); patients typically received an oral opioid alone, or an oral opioid combined with an oral benzodiazepine. The regimen of medications administered was independent of the study protocol and consisted of non-intravenous opioids (fentanyl in nearly all cases, morphine in the minority of cases, and oral benzodiazepines in the minority of cases). The opioids typically used at our burn center during this study included either oral transmucosal fentanyl lozenges (10 mcg/kg/dose to nearest available dose) or oral hydromorphone (0.05–0.1 mg/ kg), while the most commonly used procedural anxiolytic benzodiazepine was Lorazepam Dose: 0.05 mQ/kQ/dose IV/PO (Ratcliff et al., 2006; Herndon, 2018, p. 694).

**FIGURE 1 |:**
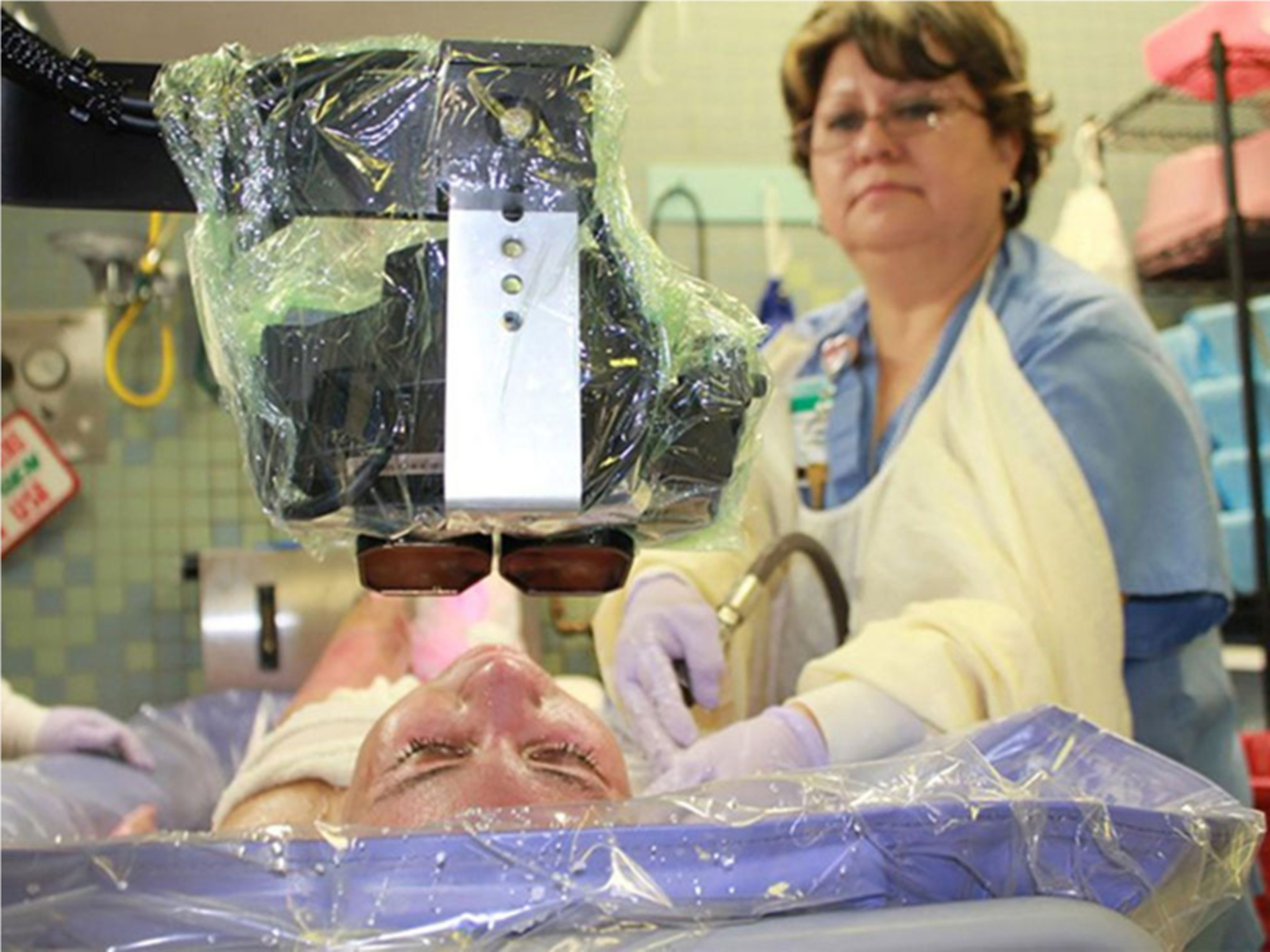
A severely burned patient looking into the VR goggles during burn wound care in the ICU tank room. Copyright Hunter Hoffman, U.W., www.vrpain.com.

The wound care sessions began with a nurse cutting off, removing, and throwing away the gauze bandages, using warm water from a hand held medical shower hose, and wet washcloths to clean debris out of the patients wounds, and applying disinfectant and fresh bandages (see Figure 1). All patients in both groups received a baseline wound care session with No VR before Study Day 1 as early as possible after their admission. During wound debridement on Study Day 1 and for up to 10 study days in total (mean = 6 study days for the No VR group), patients in the No VR group received standard of care, which involved pain medications but no virtual reality. In contrast, after 1 day of wound care with No VR during the baseline pain session, patients randomly assigned to the Yes VR group received standard of care pain medications combined with water-friendly virtual reality during their wound debridement/cleaning session on Study Day 1 for up to 10 study days in total (mean = 6 study days for the VR group). For the VR group, during wound care on study days, the research assistants placed the VR goggles weightlessly near the patient’s eyes (see Figure 1), minimizing the patients physical contact with the VR equipment (Hoffman et al., 2019b). While looking into the VR goggles, the patient used a wireless computer mouse to interact with the virtual reality world.

**FIGURE 2 |:**
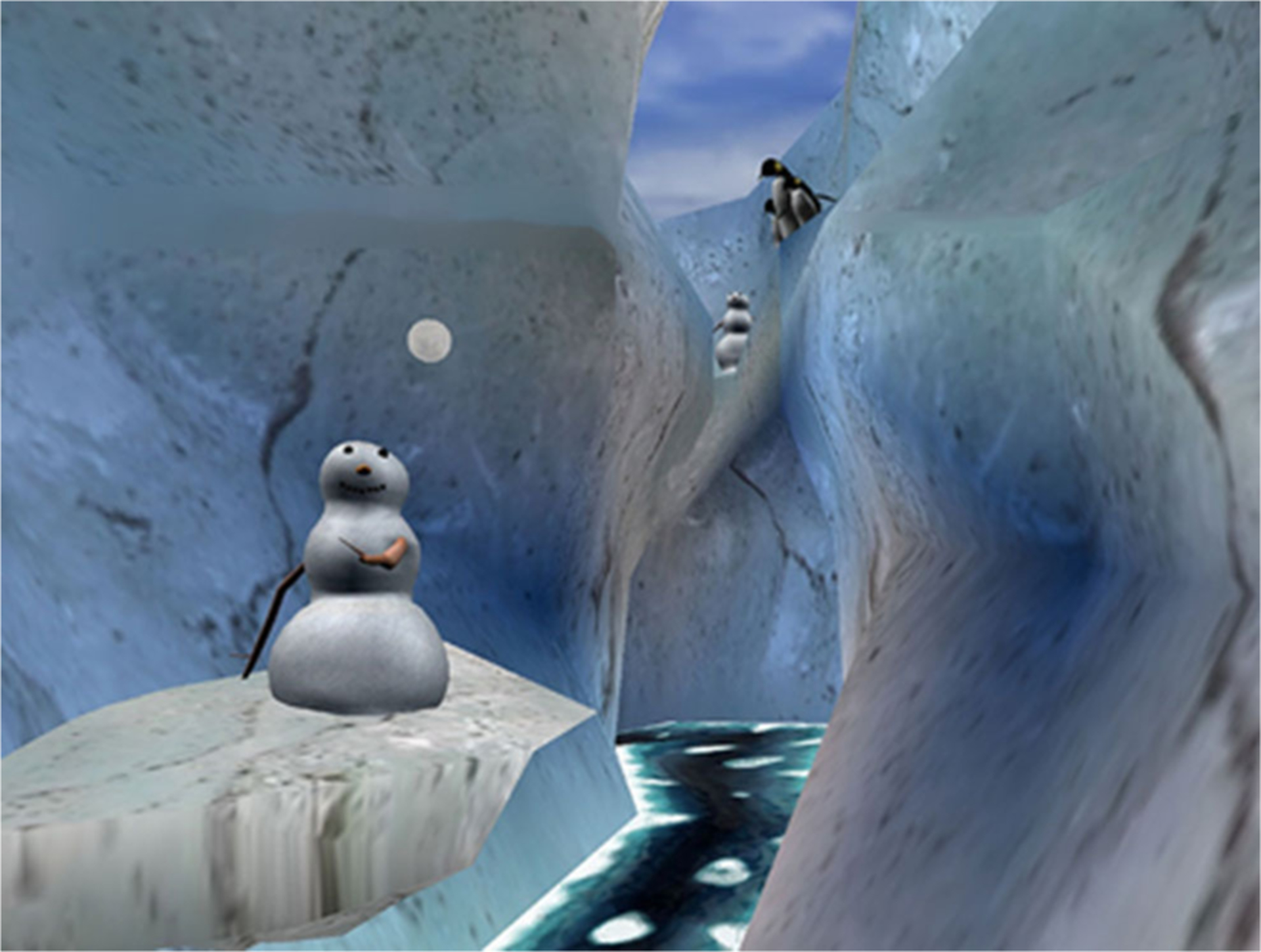
A screenshot of the virtual reality world SnowWorld, owned by the University of Washington Seattle, image by Ari Hollander and Howard Rose, copyright Hunter Hoffman, U.W., www.vrpain.com.

Patients randomly assigned to the VR group interacted with a virtual environment named SnowWorld for Study Day 1 for up to 10 study days. The original version of SnowWorld (Hoffman et al., 2001, 2004a) was the first VR world designed for pain control and was specifically designed for pediatric burn patients during painful medical procedures and physical therapy skin stretching exercises. In the newest version of SnowWorld, used in the current study (see Figure 2, www.vrpain.com), patients floated slowly through a snowy 3D virtual reality canyon they could see in the VR goggles. In the current study, the patient’s head and body remained still during wound care, and instead of moving their head to look around the virtual world (head tracking), patients used a wireless computer mouse to look around, aim and throw snowballs at snowmen, penguins, igloos, flying fish, and wooly mammoths. Subjects received converging sensory input from real time sound effects synchronized with the visual effects in VR (e.g., a mammoth raising its trunk and trumpeting angrily when hit by one of the patient’s snowballs). The sound effects were mixed with upbeat music in the background (e.g., Paul Simon’s Graceland, and a few Spanish songs).

Shortly after each burn wound debridement/cleaning session, the pediatric patients briefly answered retrospective graphic rating scale questions to measure their subjective pain ratings. The patient received fresh bandages on their burns, and the patient was returned to their room. The VR system was cleaned/disinfected and was removed from the wound care room.

### Statistical Analyses

IBM SPSS (2019) statistical analyses of the primary and secondary hypotheses involved an apriori two-tailed One Way Between Groups ANOVA, with alpha = 0.05.

## RESULTS

This study was conducted between July 2015 and December 2017. Out of 62 patients initially screened, 50 pediatric patients met our apriori inclusion criterion of having a moderate or higher “worst pain” rating during the baseline wound care session with No VR (worst pain of 5 or higher, on a zero to 10 graphic rating scale). Eighty-four percent of the patients were male, and 16% were female. Most patients in the current study had burns covering nearly half of their bodies [mean = 44% Total Body Surface Area (TBSA) burned, range 14−86% TBSA]. In other words, the smallest burn wound was 14% of his/her body (TBSA), and the patient with the largest burn was burned on 86% of his/her body. Patients’ ages ranged from 6 to 17 years of age at the time of enrollment. Sixty-eight percent of the patients had hand burns, 76% had arm burns, 29% had foot burns, 59% had leg burns, 63% had head/neck burns, 76% had trunk/torso burns, and 12% had groin burns. The etiologies of the burns were as follows: Flame = 66%, electrical = 27%, scald = 7.3%, chemical = 2%, other = 4.9%. The mean duration of wound care on baseline was 23.81 min (SD = 6.87) for the control group vs. 23.40 min (SD = 8.28) for the VR group. The mean duration of wound care on Day 1 was 24.05 min (SD = 7.35) for the control group vs. 20.20 min (SD = 7.43) for the VR group.

### Test of Primary Hypothesis

As in most or all of the previous studies by our team (e.g., see Hoffman et al., 2019a, 2020 for reviews), worst pain intensity was selected as the primary outcome measure in the current study. Worst pain is the measure most highly correlated with functional interference (Harris et al., 2007), and worst pain (sensory pain) is considered the pain measure that matters the most to the patient, the best measure of therapeutic effect.

Using a Between Groups One-Way ANOVA, for the primary outcome measure of worst pain intensity on Study Day 1, burn injured children in the group who received the custom articulated arm mounted water-friendly virtual reality treatment during wound care in the current study reported significantly less “worst pain intensity” than the No VR control group. On Study Day 1, on a 0 to 10 scale, the mean worst pain score for the No VR Control Group was 7.46 (SD = 2.93), and was 5.54 (SD = 3.56) for the Yes VR group, *F*_(1,48)_ = 4.29, *p <* 0.05, MS = 46.00, partial eta squared = 0.08, observed power = 0.53.

**FIGURE 3 |:**
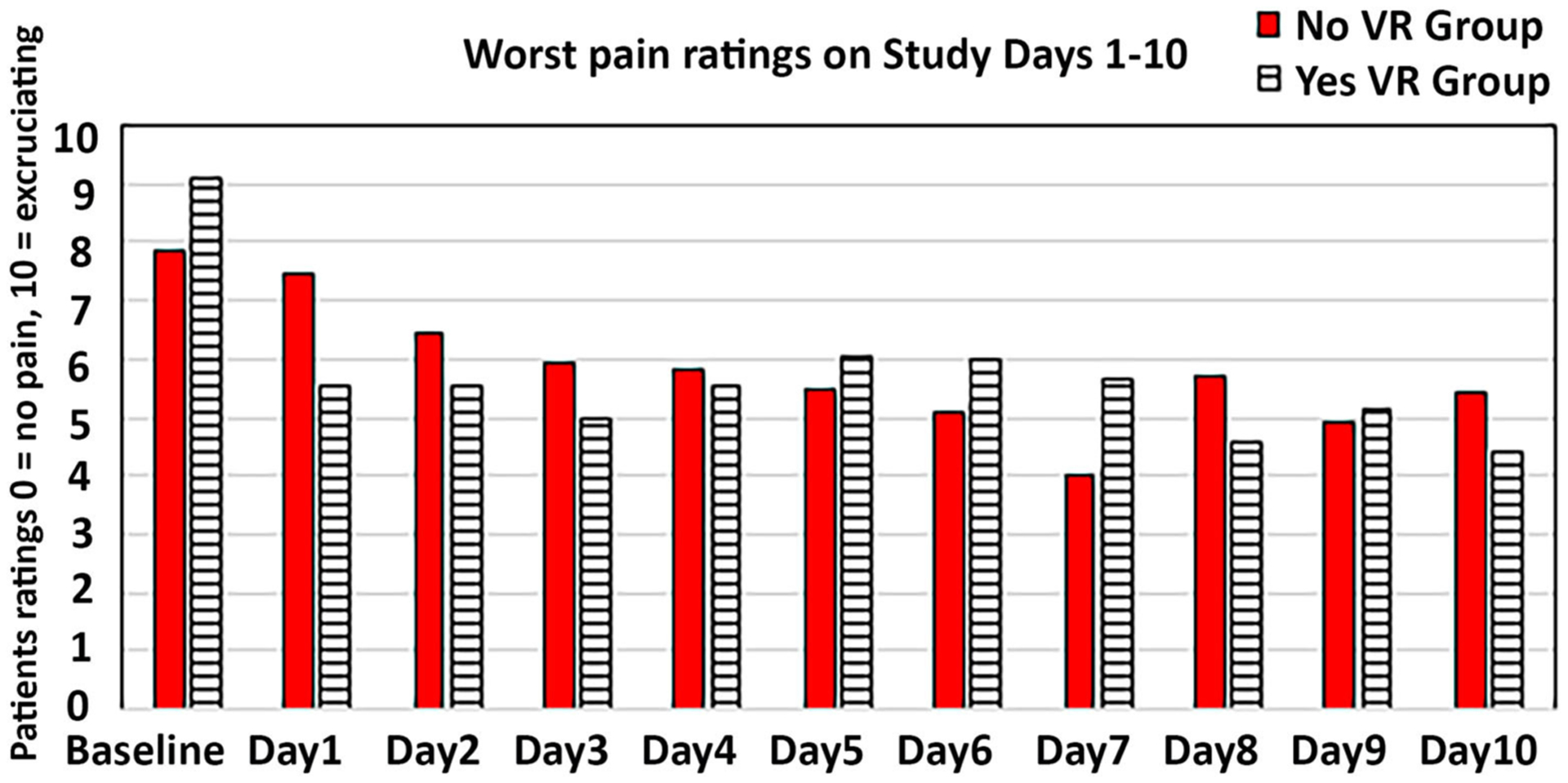
A graph showing worst pain ratings during baseline, and Study Days 1–10, for the VR Group vs. the No VR Control Group. Note that all patients received No VR during wound care on the baseline day.

In addition, absolute difference scores were calculated (baseline pain minus the mean of the worst pain scores on Study Days 1−10, see Figure 3). Absolute difference scores showed significant VR reductions in worst pain beyond Study Day 1 (where larger positive absolute difference scores indicate greater pain reduction). The mean absolute difference score for the No VR Group was 2.20 (SD = 3.06), and for the VR Group was 3.85 (SD = 2.20), *F*_(1,48)_ = 4.88, *p* < 0.05, MSE = 34.26, partial eta squared = 0.09, (a small effect size), observed power = 0.58. In other words, overall, patients in the VR group continued to show small but significant reductions in worst pain ratings, beyond Study Day 1. On average, patients in the No VR group participated for 6 study days (mean = 6.04 days, SD = 2.79), and patients in the VR group also participated for an average of6 study days with VR (mean = 5.89, SD = 3.04).

### Test of Secondary Hypothesis

Using a Between Groups One-Way ANOVA, for the secondary measure of patients ratings of “lowest pain during wound care,” (where lower is better), on Study Day 1, the No VR control group mean was 4.29 (SD = 3.75) as opposed to 1.68 during the Yes VR Group, *F*_(1,47)_ = 9.29 (MS = 83.52, *p <* 0.005, partial eta squared = 0.165, observed power = 0.85). In other words, least pain was significantly lower for the VR group. The results of the other secondary measures were non-significant but showed the predicted pattern on Study Day 1.

Time spent thinking about pain during wound care (24% lower for the VR Group), for the No VR control group mean was 6.33 (4.26) as opposed to 4.88 (3.54) during the Yes VR Group, *F*_(1,48)_ = 1.72, *p* = 0.20 NS.

Pain unpleasantness (8% lower for the VR group), for the No VR control group mean was 5.29 (3.59) as opposed to 4.96 (3.66) during the Yes VR Group, *F*_(*1,48*)_ < 1, NS.

Fun during wound care (44% higher fun for the VR group). Fun during the No VR control group mean was 2.97 (3.81) as opposed to 4.96 (3.97) during the Yes VR Group, *F*_(1,46)_ = 372, MS = 56.33, *p* = 0.06 NS, partial eta = 0.08, power = 0.47.

VR nausea was very low (< 1 on a scale from zero to 10), and subjects in the VR group reported a moderate illusion of “being there” in virtual reality and reported that the virtual objects looked moderately real.

### Did VR Continue to Reduce Pain When Used Again on Subsequent Study Days?

Using Between Group ANOVA’s comparing the No VR group vs. the VR group, absolute difference scores^1^ were non-significant for all secondary pain measures: Time spent thinking about pain, *F*_(1, 48)_ < 1, NS, pain unpleasantness, *F*_(1, 48)_ < 1, NS, Lowest pain, *F*_(1, 46)_ = 2.67, *p* = 11 NS, MSE = 18.14, partial eta squared = 0.06, pain unpleasantness, *F*_(1, 48)_ < 1, NS, and on a surrogate measure of positive emotional affect, Fun during wound care, *F*(_1, 46_) < 1, NS. One factor contributing to the finding that the primary measure was significant, and the secondary measures were not, is that there was more variance (noise) in the results of the secondary measures.

## DISCUSSION

The current study examined whether VR could help reduce pain during one of the most painful procedures in medicine. To our knowledge, this is the first randomized between-group study of children with large severe burn injuries to test whether virtual reality can reduce procedural pain. Despite the challenges of using VR in the ICU hydro tank to treat children with unusually large severe burn injuries, patients reported significant reductions in worst pain on Study Day 1 (25% less pain intensity/worst pain in the VR Group than the Control Group). In addition, the “lowest pain” the patient felt during wound care on Study Day 1 was significantly lower in the VR group (60% lower for the VR group than for the Control group). Patients in the VR group reported a moderate illusion of going inside the VR SnowWorld game, they rated the virtual objects as moderately real, and VR nausea was near zero, perhaps due in part to the lack of head movement. To summarize the secondary measure results on Study Day 1, as predicted, the secondary measure of patient’s “lowest pain” rating was significantly lower in the VR group on Study Day 1, and the results of all other secondary pain measures showed the predicted pattern of results but were non-significant on Study Day 1. The treatment duration of > 20 min for each group is an unusually long wound care duration compared to previous VR studies (e.g., Maani et al., 2011 used VR for only 6 min of wound care, and 6 min of No VR during the same wound care session). The current study had unusually long VR wound care sessions because the burn wound sizes were unusually large, and the current study used a between groups design.

In a measure of whether VR had any pain reduction benefits beyond Study Day 1, absolute difference score measures were calculated. As predicted, VR reduced pain for our primary outcome measure of worst pain during wound care. However, contrary to our predictions, absolute difference score measures were non-significant for all secondary measures (no difference between groups for time spent thinking about pain, lowest pain, pain unpleasantness, or fun during wound care, a surrogate measure of positive emotion).

In light of the current opioid overdose death epidemic (Chen et al., 2019; Wilson et al., 2020), the medical community is under growing political and medical pressure to develop and test more powerful adjunctive psychological (non-drug) pain control techniques (Keefe et al., 2018). Virtual reality is a very low risk psychological treatment, with no risk of pharmacologic overdose, no risk of over sedation, or post-anesthetic dementia, and no risk of opioid addiction associated with opioid analgesia/anesthesia. VR can help compensate for undermedication and could potentially substantially lower the opioid doses needed in some contexts (Firoozabadi et al., 2020). The current study is an important step in the direction of how VR could be used in clinical practice in the ICU, in particular. Although preliminary, the current results add to evidence from the pilot study by Hoffman et al. (2019b) that water-friendly virtual reality can reduce pain intensity (worst pain ratings) during painful medical procedures. The portable wide field of view VR goggle system with a robot-like articulated arm goggle holder used in the current study was customized to allow treatment of severely burned children who could not wear a VR helmet on their heads.

Slater and Wilbur (1997) identified several factors that contribute to the immersiveness of a VR system. According to Slater and Wilbur (1997), field of view of the VR goggles, and interactivity (being able to interact with objects in the virtual world) are two important elements of an immersive VR system. At the time data collection for the current study began in 2015, with the exception of studies that used SnowWorld (typically with relatively wide field of view VR helmets) most non-SnowWorld VR pain distraction studies used much less expensive, narrow (28 to 35 degree diagonal) field of view, low resolution VR helmets (e.g., Sil et al., 2014).

At the time that this study began in 2015, the portable water-friendly VR system used in the current study had exceptionally large wide field of view military grade VR goggles (90 degree diagonal field of view, with relatively high resolution by pre-Oculus standards, and high quality optics, the current study used military grade, $35,600 goggles). This extra peripheral vision of the MX90 VR goggles was designed to increase the patients illusion of presence (Prothero and Hoffman, 1995), to make VR more distracting, in light of concerns that it may be difficult to distract children with large severe burns during wound care, who are in such intense pain, and to help compensate for the absence of head tracking due to the robot-like arm goggle holding system. Similarly, the software used in the current study, SnowWorld, allowed patients to interact with objects in the virtual world during wound care, to help increase their illusion of presence. SnowWorld was specifically designed by our team for patients who were highly medicated, and in extreme pain, who would likely not be able to play a more complicated conventional video game during wound care. In previous laboratory studies, wide field of view (Hoffman et al., 2006), and interactivity (Hoffman et al., 2004b; Dahlquist et al., 2007; Wender et al., 2009; Al-Ghamdi et al., 2020) have been shown to significantly increase VR pain reduction. The essential core quality of virtual reality is participant’s illusion of “being there” in the computer-generated environment, as if the VR world is a place the person visited (Slater and Wilbur, 1997). In the current study, participants rated their illusion of presence in VR as a “moderate sense of going inside,” despite the immobilized VR goggles (no head tracking) and extreme pain levels.

## LIMITATIONS

The current study had a number of limitations that should be taken into consideration when interpreting the results. All of the 50 pediatric patients in the current study were children, and most (88%) were from Latin America, and spoke Spanish, and the current study focused exclusively on severely burned children, which further limits generalizability of the current findings. The water friendly VR system used in the current study was custom designed for patients with head and face burns. Patients could not be blind to (i.e., unaware of) the research demands within the VR condition. The children were likely able to directly connect study questions to the use of VR equipment. Future randomized controlled between group research studies are needed, ideally blinding patients to treatment conditions, to reduce bias (Schulz and Grimes, 2002; Houle, 2015). One possibility is to use multiple conditions that use similar equipment but vary with respect to features (e.g., an interactive condition vs. a passive observer condition).

The water-friendly VR system used in the current study was customized to minimize or ideally eliminate physical contact between the VR goggles and the burn patient. Patients used mouse tracking to look around and throw snowballs. However, the patients could not move their heads around to look around in virtual reality like they could with a conventional head tracked VR helmet. For this reason, one limitation of the current VR system is that, although having a wide field of view and being highly interactive, the water-friendly VR system was not fully immersive (no head tracking).

Another important limitation is that the current study does not explore why VR reduces patients’ pain. The logic of why VR reduces pain often assumes an attentional mechanism (Hoffman et al., 2000a,b; Birnie et al., 2017). However, the mechanism of action within the VR condition is not well-understood. Several related mechanisms are likely involved. The VR environment can engender a positive affect (as evidenced in the current study by the significant increase in “fun,” a surrogate measure of positive affect), and the VR environment can evoke pleasant memories, e.g., SnowWorld may evoke memories of Christmas, snow skiing and other happy memories. The current study did not test whether VR relies on an attentional mechanism—another limitation. Future studies that elucidate the mechanism of action are needed. A better understanding of the mechanism of how VR reduces pain may increase our understanding of how VR achieves therapeutic effects and may inform the design of future virtual reality pain reduction systems. However, to increase statistical power, for clinical studies involving active control conditions, we would recommend a within-subject, within wound care design (with treatment order randomized) and a large sample size for future studies comparing the effectiveness of more than one treatment condition in a clinical trial (e.g., a “wide field of view +interactive VR” Group vs. a passive VR Group vs. a No VR group).

### Strengths

In spite of these limitations, the current study makes several important contributions to the literature, with important implications for clinical practice. This is the first randomized between group study to measure VR pain reduction in the ICU, in predominantly Latino pediatric patients with unusually large severe burn injuries. The current study replicates our recent previous pilot findings that children with large severe burns were generally able to engage in a computer generated world during severely painful medical procedures, and results support our previous results showing that VR significantly reduced patients’ worst pain ratings during wound care (Hoffman et al., 2019b). These results address the important practical question of whether it is possible to use VR in this challenging context, and whether virtual reality can help reduce the amount of pain experienced by children during burn wound cleaning sessions. The current results show it is feasible to use VR pain reduction in this challenging patient population and medical context, with little or no VR side effects.

## FUTURE DIRECTIONS

Although the VR group reported significantly less pain intensity (worst pain ratings) during wound care, the lack of significant results on most of the secondary measures indicate that a stronger dose of VR is needed. Additional research and development of more effective VR pain reduction systems is recommended. Improvements to the VR system to increase the illusion of presence could make the VR system more immersive, and we predict, can significantly further increase how much pain is reduced. More immersive VR systems with a wider field of view (Hoffman et al., 2006), increased interactivity (Hoffman et al., 2004b;Dahlquist et al., 2007; Wender et al., 2009), eye tracking (Al-Ghamdi et al., 2020), and tactile feedback (Hoffman et al., 1998) may help make VR more effective in the future (Hoffman et al., 2020). In addition, more effective pain medications (McIntyre et al., 2016), and a better understanding of how to integrate pharmacologic and non-pharmacologic analgesics most effectively are important directions for future research. Virtual reality (VR) may reduce the opioid analgesic doses needed by patients during painful medical procedures (Kipping et al., 2012; McSherry et al., 2018). In other situations (e.g., children suffering such excruciating pain during No VR that they are unable to play SnowWorld) increasing the opioid dose may lower patients pain enough that the patient is able to play VR, further reducing the patients pain during medical procedures.

### VR May Help Prepare Patients Before Wound Care

Burn patients often become anxious before their wound care sessions. They ruminate about previous painful wound care sessions and anticipate that the wound care session they are about to receive is going to be painful. They may catastrophize or have exaggerated fears about how much pain they will experience during wound care. Future studies should explore the use of virtual reality during the 25 min before the patient’s wound care. VR may help reduce pre-procedure anxiety and common negative thoughts and emotions, so that patient enter the wound care session in a healthier psychological state of mind and may thus experience less pain during their wound care session.

Due to the investment of large corporations into mass marketing of VR hardware for entertainment, this technology has recently become more immersive, more affordable, and there is an increasing demand for dissemination of effective treatments (Bailenson, 2018; Frist, 2018; Hoffman et al., 2020). VR has the potential to help improve the medical and psychological outcome of patients undergoing burn wound care, which could reduce reliance on opioids in some patients, and could significantly reduce healthcare costs. Additional research and development of more immersive (more distracting) VR systems is recommended.

## Footnotes

1 Absolute difference = baseline minus mean of each patient’s worst pain ratings on up to 10 study days.
